# Inflammatory demyelinating polyneuropathy versus leptomeningeal disease following Ipilimumab

**DOI:** 10.1186/s40425-018-0318-x

**Published:** 2018-01-30

**Authors:** Lorraine Cafuir, David Lawson, Nilesh Desai, Vita Kesner, Alfredo Voloschin

**Affiliations:** 1 Department of Hematology and Medical Oncology, Winship Cancer Institute of Emory University, 1365C Clifton Road NE, Suite C5010, Atlanta, GA 30322 USA; 2 Department of Radiology, Baylor College of Medicine, 6701 Fannin Street, Suite 470, Houston, TX 77030 USA; 3 Department of Neurology, Emory University School of Medicine, 12 Executive Park Drive NE, Atlanta, GA 30329 USA

**Keywords:** Ipilimumab, Melanoma, Leptomeningeal carcinomatosis, Inflammatory demyelinating polyneuropathy, Autoimmune, Immunotherapy, Paraneoplastic autoimmune disease

## Abstract

**Background:**

Ipilimumab is an FDA-approved anti-CTLA-4 monoclonal antibody used in treatment of metastatic melanoma. We present an unusual neurological complication of Ipilimumab therapy and the diagnostic dilemma it caused.

**Case presentation:**

A 42 year old male with Stage IV metastatic melanoma developed lower extremity weakness and sensory neuropathy following three doses of Ipilimumab. MRI of the lumbar spine was initially interpreted as diffuse leptomeningeal disease, and patient began Dexamethasone and radiation with improvement in symptoms. However, subsequent completion imaging revealed smooth nerve root involvement with sparing of the spinal cord, findings more compatible with inflammatory demyelinating polyneuropathy. The absence of malignant cells in the cerebrospinal fluid (CSF) and nerve conduction study (NCS) showing lumbar polyradiculoneuropathy with axonal involvement and demyelinating features supported the diagnosis of inflammatory demyelinating polyneuropathy. Later in the course of his disease, the patient developed frank leptomeningeal melanoma.

**Conclusion:**

Ipilimumab immune-related toxicity presented as inflammatory demyelinating polyneuropathy, which was difficult to distinguish from leptomeningeal disease, a common complication of melanoma.

## Background

The frequency of leptomeningeal metastatic spread of melanoma in stage IV patients is 22–46% [1]. Autoimmune neurologic events are a recognized complication of Ipilimumab therapy. Melanoma is associated with paraneoplastic neurologic syndromes. Determining the etiology of neurological complications in a patient with melanoma may thus present a challenge. It is important that the correct diagnosis is made because treatments are different and increasingly effective. We present a patient who developed inflammatory demyelinating polyneuropathy while taking Ipilimumab along with Vemurafenib for systemic disease, who was successfully treated with corticosteroids and discontinuation of Ipilimumab. Interestingly, he later developed clear leptomeningeal disease as part of his overall disease progression.

## Case presentation

A 42 year old Caucasian male underwent wide local excision of a truncal 3.05 mm non-ulcerated melanoma with nevoid and spitzoid features. Sentinel node biopsy was negative. Two years later, a palpable left groin nodule was treated with superficial inguinal and deep pelvic lymphadenectomy followed by Interferon therapy. Interferon was tolerated poorly, with headaches and confusion, and stopped in the first month of therapy. Two and a half years later, PET/CT showed diffuse metastatic disease to the lungs, liver, right adrenal, and hilar lymph nodes. He was enrolled in a study evaluating 6 weeks of Vemurafenib followed by Ipilimumab 10 mg/kg (higher than the FDA approved dose of 3 mg/kg) intravenously every 3 weeks (NCT01673854) [2]. After 6 weeks of Vemurafenib, tumors had shrunk by 9%. Ipilimumab was initiated 8 weeks after his first dose of Vemurafenib. After the second dose of Ipilimumab, he developed Grade 1 fatigue and began having intermittent mild headaches attributed to stress at work. MRI of the brain was negative.

**Fig. 1:**
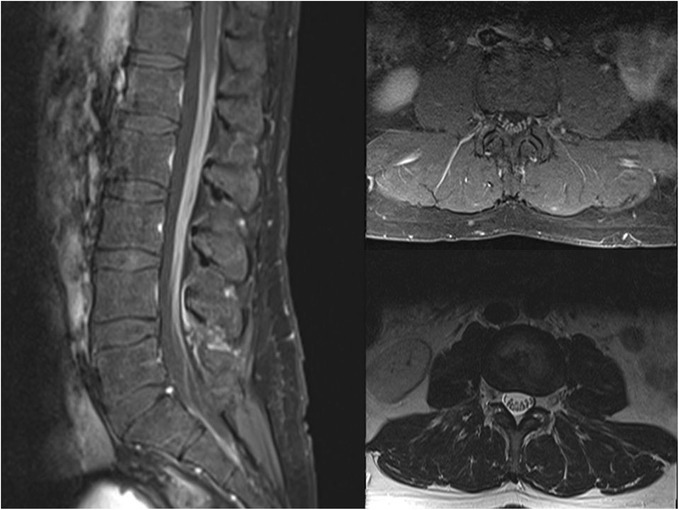
Initial MRI lumbar spine following Ipilimumab. Sagittal and axial T1w fat (left and top right) saturated post contrast and axial T2w FSE (bottom right) images demonstrating smooth, non-nodular avid enhancement of cauda equina without significant nerve root thickening

Six days following the third dose of Ipilimumab, he presented with bilateral hip pain 8 on a 10 point scale, subjective bilateral thigh weakness, and paresthesia of the soles of his feet. Initial examination showed normal muscle strength except for the left quadriceps (4/5). Upper extremity strength was intact. Sensory exam was not performed. MRI of the lumbar spine demonstrated diffuse enhancement surrounding the entire conus and all of the nerve roots thought most compatible with leptomeningeal carcinomatosis (Fig. 1). The remainder of the spine was not imaged because of the patient’s pain and absence of signs/symptoms suggestive of involvement above the lumbar spine. PET/CT performed the following day showed 23% increase in extra-CNS tumor measurements. Vemurafenib was resumed because of worsening of disease on Ipilimumab and previous improvement, although slight, on Vemurafenib for 6 weeks.

**Fig. 2:**
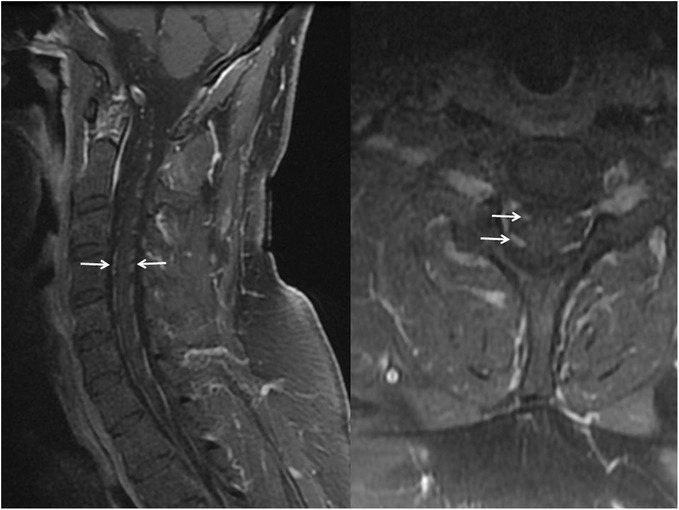
Follow up post contrast cervical spine MRI. Parasagittal and axial T1w fat saturated post contrast images demonstrating smooth avid enhancement of all anterior and posterior cervical nerve roots (arrows) but no involvement of the cord surface. Both factors strongly mitigate against leptomeningeal metastatic disease

Patient’s lower extremity weakness, pain, and numbness worsened rapidly over the next week with deterioration to ECOG 3. There were still no cranial nerve nor upper extremity signs nor symptoms. Vemurafenib was held, dexamethasone was started, and daily radiation was initiated to conus and cauda equina at 3 Gy per fraction for presumed leptomeningeal disease. However, completion MRI of the spine done after 5 days of radiation, and 16 days after the initial MRI of the lumbar spine, revealed diffuse, symmetric, and smooth nerve root enhancement affecting the entire spine while sparing the entirety of the cord surface; imaging findings more compatible with inflammatory demyelinating polyneuropathy (Fig. 2). Brain MRI was normal. Radiation was stopped, neurology was consulted, and workup for inflammatory demyelinating polyneuropathy was initiated. CSF was negative for malignant cells, but showed significantly increased protein without pleocytosis (Table 1). Autoimmune testing was unremarkable (Table 1). Electrophysiological studies were done 8 weeks after the onset of the first neurological complaints, and at that point, his neurological exam showed weakness in foot dorsiflexion, knee and hip flexion bilaterally, worse on the left. He had impaired vibration in the toes and absent delayed tendon reflexes in the lower extremities.

**Table 1:** Neuro-oncology evaluation

Laboratory test	Value	Normal	Units
Blood			
TSH	2.11	0.55–4.78	mcIU/mL
CK	91	49–397	Unit/L
WBC	11.7	4.2–9.1	K/uL
Vitamin B12	587	211–911	Pg/mL
CSF			
Initial			
WBC	1	0–5	Cells/uL
RBC	0	0	Cells/uL
Protein	> 300	15–45	Mg/dL
Glucose	73	40–70	Mg/dL
Cytology	Negative for malignant cells		
6 month Follow-up			
WBC	3	0–5	Cells/uL
RBC	6	0	Cells/uL
Protein	169	15–45	Mg/dL
Glucose	56	40–70	Mg/dL
Cytology	atypical cells with hyperchromatic nuclei and pigmentation, suspicious for melanoma		
Autoimmune Studies			
Asialo-GM1 (IgG/IgM)	13	0–50	IV
GM2 (IgG/IgM)	4	0–50	IV
GD1a (IgG/IgM)	8	0–50	IV
GD1b (IgG/IgM)	4	0–50	IV
GQ1b (IgG/IgM)	5	0–50	IV
GM-1 (IgG)	3	0–50	IV
GM-1 (IgM)	5	0–50	IV
AMA	1.5	0–20	Units
Rheumatoid Factor	< 20	< 20	IU/mL
ANCA	< 1:20	< 1:20	
dsDNA antibody	Negative		
ENA	Negative		
Borrelia burgdorferi antibody	0.20	0–1.20	LIV
Leishmania antibody IgG	0	0	units
Hepatitis panel			
Hepatitis B surface antigen	Negative		
Hepatitis B surface antibody	0.04^a^		mIU/mL
Hepatitis B core antibody	Negative		
Hepatitis C antibody	Negative		
Viral studies			
CMV antibody	Positive		
EBV nuclear antigen	Positive		
EBV viral capsid antigen IgG	Positive		
EBV viral capsid antigen IgM	Negative		
HIV	Negative		
Herpes 1 IgG	Negative		
Herpes 2 IgG	Negative		

*Abbreviations*: *TSH* thyroid stimulating hormone, *CK* creatinine kinase, *WBC* white blood cell, *RBC* red blood cell, *GM* monosialoganglioside, *GD* disialoganglioside, *GQ* tetrasialoganglioside, *ENA* extractable nuclear antigen screen, *ANCA* anti-neutrophil cytoplasmic antibody, *dsDNA* double stranded DNA, *AMA* Mitochondrial M2 antibody IgG, *CMV* Cytomegalovirus, *EBV* Epstein-Barr virus, *HIV* human immunodeficiency virus, *IV* index value, *LIV* Lyme index value

^a^Hepatitis B surface antibody < 8 mIU/mL indicates inadequate antibody response to vaccination

NCS were normal in the upper extremities despite abnormal cervical spine MRI but showed multiple abnormalities in the lower extremities including absent sensory response in the right sural nerve, markedly slowed conduction velocities between the ankle and below the fibular head stimulation, severely reduced compound muscle action potential (CMAP) amplitudes, and mild-to-moderate prolongation of the distal latency on the left side. Left tibial nerve motor NCS showed significantly prolonged distal latency with moderately decreased CMAP amplitudes and slowed conduction velocity. Right tibial nerve motor NCS was normal except for a mild prolongation of distal latency and mildly slowed conduction velocity. The F-wave responses of the bilateral tibial nerves were absent, indicating a more proximal conduction block. Needle electromyography of the left lower extremity was significant for denervation changes in lumbar paraspinal and tibialis anterior muscle as well as neurogenic changes in all the tested muscles of the right leg. The study was indicative of an asymmetric, subacute to early chronic and ongoing lumbar polyradiculoneuropathy with axonal involvement and demyelinating features. It is not clear why the cervical and thoracic spine findings did not cause detectable signs nor symptoms.

Vemurafenib was restarted and Dexamethasone was continued. Patient had partial response systemically and neurologic improvement. Dexamethasone was tapered and then stopped almost 6 months after initiation. It is not possible to evaluate the extent to which the dexamethasone or anti-melanoma agents contributed to this outcome.

PET/CT 6.5 months after resuming Vemurafenib showed progression of disease. Brain MRI showed multiple new foci of nodular leptomeningeal enhancement consistent with metastases. CSF cytology was concerning for metastases (Table 1). Vemurafenib was discontinued, and intrathecal IL2 and Dabrafenib therapy was initiated at another institution. Head CT after 5 months of intrathecal IL2 showed progression with the disease now predominantly dura-based, and patient elected for home hospice.

## Discussion

Twenty-two to 46% of patients with stage IV melanoma have leptomeningeal involvement by the disease. Conversely, inflammatory demyelinating polyneuropathy presenting as paraneoplastic autoimmune disease associated with melanoma independent of immunotherapy is extremely rare, with only 10 cases reported in literature to date [3, 4], and maybe due to shared immunogenic ganglioside antigens [5] or to infectious and other agents associated with these neuropathies when they are seen in the absence of associated malignancy or immunotherapy. Case reports of sensorimotor neuropathy following Ipilimumab treatment [6–8] describe a variety of syndromes including CIDP [7], multifocal polyradiculoneuropathy [8], and meningo-radiculo-neuritis [6]. The neurologic complications in our patient are consistent with those seen in patients who received Ipilimumab alone but have not to our knowledge been reported with Vemurafenib.

Patients with melanoma who develop neurologic complaints compatible with disease involving the spinal cord are most likely to have MRI of the spine as the first and frequently only diagnostic workup. Depending on the burden of metastatic disease, leptomeningeal carcinomatosis of the spine can have variable appearance. With mild disease, smooth, contiguous or noncontiguous fine coating of the cord surface and nerve roots, termed “sugar coating” or “zuckerguss” can be seen [9]. Discrete nodules, large or small or even long segments of bulky mass-like disease can be seen in more severe disease. In either case, for the entity to cause diffuse, non-nodular involvement of cervical, thoracic and lumbar nerve roots, sparing the cord surface entirely, would be atypical for leptomeningeal carcinoma but very compatible with an inflammatory demyelinating polyneuropathy. The finding of non-nodular involvement of nerve roots without involvement of the cord in a patient receiving Ipilimumab or other immunomodulatory agents should prompt further evaluation for an immune based mechanism, especially since steroids or other therapies may significantly alter the course. Standard CSF studies across these cases, apart from negative cytology, have variable appearance, from normal to albuminocytologic dissociation to pleocytosis. They are not likely to be diagnostic beyond helping to rule out leptomeningeal carcinomatosis. Autoimmune workup in our patient was negative. The most helpful studies were NCS [10], as is usually the case in cases of inflammatory demyelinating polyneuropathy not associated with malignancy. Based on these findings, we would recommend that patients with cancer who were treated with immunotherapy and presenting with sensory and motor weakness for whom there is suspicion on MRI of an inflammatory demyelinating polyneuropathy, should undergo a thorough neurological evaluation which includes NCS, CSF studies, and consultation with a neurologist.

## Conclusion

Leptomeningeal carcinomatosis and inflammatory demyelinating polyneuropathy, whether idiopathic or related to Ipilimumab or other immunomodulatory agents, may have similar clinical presentation, making it difficult to distinguish them. MRI is usually the initial study in patients presenting with signs/symptoms of spinal involvement. The finding of involvement of nerve roots, usually in a smooth, non-nodular fashion, with sparing of the surface of the cord should raise suspicion of an autoimmune, inflammatory process. Since immunomodulatory treatment may improve the course of this condition, more aggressive evaluation, such as NCS and CSF studies and evaluation by a neurologist are recommended. Inflammatory demyelinating polyneuropathies whether causally related to malignancy, immunotherapy, or not are complex and recommended treatments differ. Further studies in this area are clearly warranted.

## References

[1] Le Rhun E, Taillibert S, Chamberlain MC (2013). Carcinomatous meningitis: Leptomeningeal metastases in solid tumors. Surg Neurol Int.

[2] Amin A, Lawson DH, Salama AK, Koon HB, Guthrie T, Thomas SS, O'Day SJ, Shaheen MF, Zhang B, Francis S, Hodi FS (2016). Phase II study of vemurafenib followed by ipilimumab in patients with previously untreated BRAF-mutated metastatic melanoma. J Immunother Cancer.

[3] Chau AM, Yu A, Keezer MR (2013). Chronic inflammatory demyelinating polyneuropathy and metastatic melanoma. Can J Neurol Sci.

[4] Dbouk MB, Nafissi S, Ghorbani A (2012). Chronic inflammatory demyelinating polyneuropathy following malignant melanoma. Neurosciences (Riyadh).

[5] Noronha AB, Harper JR, Ilyas AA, Reisfeld RA, Quarles RH (1986). Myelin-associated glycoprotein shares an antigenic determinant with a glycoprotein of human melanoma cells. J Neurochem.

[6] Bompaire F, Mateus C, Taillia H, De Greslan T, Lahutte M, Sallansonnet-Froment M, Ouologuem M, Renard JL, Gorochov G, Robert C, Ricard D (2012). Severe meningo-radiculo-neuritis associated with ipilimumab. Investig New Drugs.

[7] Liao B, Shroff S, Kamiya-Matsuoka C, Tummala S (2014). Atypical neurological complications of ipilimumab therapy in patients with metastatic melanoma. Neuro-Oncology.

[8] Manousakis G, Koch J, Sommerville RB, El-Dokla A, Harms MB, Al-Lozi MT, Schmidt RE, Pestronk A (2013). Multifocal radiculoneuropathy during ipilimumab treatment of melanoma. Muscle Nerve.

[9] Kale HA, Sklar E (2007). Magnetic resonance imaging findings in chronic inflammatory demyelinating polyneuropathy with intracranial findings and enhancing, thickened cranial and spinal nerves. Australas Radiol.

[10] Allen JA, Ney J, Lewis RA. Electrodiagnostic errors contribute to chronic inflammatory demyelinating polyneuropathy misdiagnosis. Muscle Nerve. 2017. [Epub ahead of print]. 10.1002/mus.25997. 10.1002/mus.25997 29053880

